# Effects of Supplemental Oxygen on Maternal and Neonatal Oxygenation in Elective Cesarean Section under Spinal Anesthesia: A Randomized Controlled Trial

**DOI:** 10.1155/2014/627028

**Published:** 2014-02-20

**Authors:** Arunotai Siriussawakul, Namtip Triyasunant, Akarin Nimmannit, Sopapan Ngerncham, Promphon Hirunkanokpan, Sasiwalai Luang-Aram, Nusaroch Pechpaisit, Aungsumat Wangdee, Pornpimol Ruangvutilert

**Affiliations:** ^1^Department of Anesthesiology, Faculty of Medicine, Siriraj Hospital, Mahidol University, Bangkok 10700, Thailand; ^2^Office for Research and Development, Faculty of Medicine, Siriraj Hospital, Mahidol University, Bangkok 10700, Thailand; ^3^Department of Pediatrics, Faculty of Medicine, Siriraj Hospital, Mahidol University, Bangkok 10700, Thailand; ^4^Department of Obstetrics and Gynaecology, Faculty of Medicine, Siriraj Hospital, Mahidol University, Bangkok 10700, Thailand

## Abstract

The use of supplemental oxygen in uncomplicated cesarean deliveries under spinal anesthesia has been thoroughly investigated during recent decades. The aim of this study was to determine the benefits for both mother and infant of administering supplemental, low-dose oxygen via a nasal cannula versus having no supplement (i.e., room air only). Healthy parturients at term undergoing elective cesarean section under spinal anesthesia were randomly allocated into two groups: an oxygen group (*n* = 170), who received 3 LPM oxygen via a nasal cannula; and a room-air group (*n* = 170), who were assigned to breathe room air. Maternal oxygen saturation was measured continuously by using pulse oximeter. The desaturation was determined by oxygen saturation <94% over 30 seconds. Umbilical cord gases and Apgar scores were collected followed delivery of the infant. All maternal desaturation events occurred in 12 parturients assigned to the room-air group. Most events were concurrent with hypotension. The umbilical venous partial pressure of oxygen was significantly higher in the oxygen group. The other blood gas measurements and Apgar scores were not significantly different between the two groups. Based on our findings, the use of supplemental oxygen could prevent maternal desaturation resulting from receiving sedation and intraoperative hypotension.

## 1. Introduction

The use of supplemental oxygen for parturients undergoing cesarean section under spinal anesthesia has been routine practice for more than 30 years [1]. Many studies in the 1980s demonstrated that maternal hyperoxia increased fetal partial pressure of oxygen and improved acid base status [2, 3]. In addition, the Royal College of Anesthesiology of Thailand suggests that supplemental oxygen should be administered via a nasal cannula or a face mask while conducting a spinal anesthesia [4]. Since adequate sensory block for the operation should cover the fourth thoracic dermatome, deterioration in pulmonary function occurs concurrently. Significant reduction in peak expiratory flow, forced vital capacity, forced expiratory volume in one second, and forced expiratory flow in the midregion of the forced vital capacity were demonstrated following spinal anesthesia in a supine position [5]. Parturients were also at risk of hypotension after the sympathetic blockade. Any of these conditions might cause fetal acidemia [6]. As a result, the use of supplemental oxygen in all elective cesarean sections was indicated in clinical practice for fetal well being.

Supplemental oxygen via a low flow oxygen system, that is, providing oxygen via a nasal cannula at 3 liters per minute (LPM), is felt to be acceptable in our practice. Nasal cannulae are more likely to remain in position than face masks because they can be better tolerated by patients and they can remain in situ in the case of vomiting. However, many questions on the benefits of routinely administering low-dose supplemental oxygen have arisen. As for the maternal aspect, oxygen therapy is currently recommended when parturients suffer from a major trauma, sepsis, or an acute illness. The target of oxygen saturation monitored by pulse oximetry is 94–98%. What is the evidence that it is appropriate to provide oxygen even though the mother is not hypoxemic? [7] As for the neonatal aspect, maternal use of inspired oxygen fractions <0.5 did not cause any change in the partial pressure of oxygen in the umbilical vein [2, 8]. Therefore, we conducted a prospective, randomized, controlled trial to determine the benefits of routinely administering supplemental, low-dose oxygen to uncomplicated parturients undergoing elective cesarean section under spinal anesthesia. The primary outcome of the study was desaturation in parturients, and the secondary outcomes were cord gases analysis and Apgar scores of the neonate.

## 2. Methods

The study protocol was approved by the Institutional Review Board. Informed consent was obtained from all participants in this study. The protocol was registered at Clinical-Trial.gov (registration number NCT01530971). The study included a total of 340 consecutive adult parturients with term singleton pregnancy who were undergoing elective cesarean section under spinal anesthesia. Parturients were excluded if they had any one of the following conditions: preoperative hypoxemia (oxygen saturation <94%), diabetes mellitus, hypertension, heart disease, obesity, placenta previa, premature rupture of membranes, and intrauterine growth retardation.

Allocation of parturients was done by simple randomization from computer-generated random numbers, and the randomization list was prepared by using the opaque, sealed envelope technique by an independent person not involved in the trial. On arrival at the operating theatre, 300–500 mL of isotonic solution was given prior to induction of spinal anesthesia. Standard monitoring included continuous electrocardiography and pulse oximetry. Noninvasive blood pressure was measured at one minute intervals from one minute after spinal injection until delivery. Subsequently, the blood pressure was measured every 5 minutes until the end of all procedures. Parturients were then randomly allocated either to breathe oxygen 3 LPM via a nasal cannula (the oxygen group) or not to receive oxygen (the room-air group). A nasal cannula was immediately applied to the oxygen group before the spinal anesthetic was given. Spinal anesthesia was performed with a 25 G or 27 G Quincke needle or a pencil point needle, with the parturients in the lateral position. The space between either the second and third or the third and fourth lumbar vertebrae was used. Spinal anesthesia was established with 0.5% hyperbaric bupivacaine and morphine. The parturients were then positioned supine with a 15 cm left lateral tilt. Anesthetic level was achieved at the fourth thoracic dermatome, tested by cold sensation or pinprick. Incremental doses of ephedrine or norepinephrine were given intravenously to maintain a systolic arterial pressure of greater than 90 mm Hg or 20% of the baseline values. Desaturation was defined by a reduction of oxygen saturation (SpO_2_) below 94% for more than 30 seconds, confirmed by good signal quality and no probe displacement. This is a modification from the recommendation from O'Driscoll et al. [7] and Van de Louw et al. [9]. If desaturation was detected, the patients were treated promptly with supplemental oxygen to keep SpO_2_ >94%.

Immediately after birth, and while the placenta was still in situ and before the infant's first breath, double clamps were placed on the umbilical cord. The umbilical artery and venous blood were collected into heparinized syringes. Each sample's oxygen partial pressure (PaO_2_), carbon dioxide partial pressure (PaCO_2_), oxygen saturation, pH, and bicarbonate were measured using a blood gas analyzer (STAT Profile pHOXPlusL; NovaBiomedical, Waltham, MA). The Apgar scores of the neonate at one and five minutes were noted by an assessor who was blinded to the study protocol.

*Statistical Analysis.* The sample-size calculation was based on the assumption that oxygen administration would reduce the incidence of desaturation from 5% to almost none (i.e., 0.1%). One hundred and sixty-two parturients were needed in each group in order to have 80% power to detect the difference with a significance level of 0.05 (two-sided hypothesis). The incidence of desaturation of 5% in the room-air parturient group was postulated from a previous study [10]. The sample size was inflated by 10% to account for possible incomplete information in records; therefore, 170 subjects were needed in each group. All analyses would be based on an intention-to-treat principle. Descriptive statistics were used to examine the preoperative characteristics and the perioperative variables. The proportion of desaturation was compared, using the Chi-square test. Continuous, normally distributed variables were compared with the Student's *t*-test, and for nonnormal distributed, the Mann Whitney *U*-test was used. Statistical analysis was conducted using a software program, SPSS version 18, SPSS Inc., Chicago, IL, USA. Data was presented as a mean ± standard deviation (SD) or a number (percent) or 95% CI, as appropriate. *P* < 0.05 (2-sided) was considered to indicate statistically significant differences.

## 3. Results

A total of 340 parturients were enrolled in the study. Fifteen participants were excluded from the study because they had labor pain and required emergency cesarean section. The mothers' ages, body mass index, baseline hematocrit, and indications for cesarean section for the two groups were similar. The surgical details, including uterine incision to delivery interval, duration of surgery, and estimated blood loss, were also similar between the groups. In addition, the amount of preload fluid, total intraoperative fluid administration, and vasopressor consumption were not significantly different. However, maternal hypotension occurred more frequently in the room-air group than the oxygen group (81% versus 69.8%, *P* = 0.01). As for the primary outcome of this study, the proportion of desaturation was significantly different between the groups, and all events occurred in the room-air group (7.4% versus 0%, *P* = 0.00) (Table 1).

Data for the 12 parturients who experienced intraoperative desaturation is shown in Table 2. Nine out of twelve events were attributed to intraoperative hypotension, and three to the sedative administration. All pronounced desaturations were treated with oxygen via a cannula or mask to keep saturation >94%. As for those parturients who experienced desaturation concurrent with hypotension, appropriate responses were increasing blood pressure, using bolus ephedrine or norepinephrine, and loading intravenous fluid. Hypotension from high spinal block was managed with the head-tilted supine position. In the case of sedated patients with desaturation, they were easily treated with oxygen and head positioning.

The neonatal oxygen data and outcomes are summarized in Table 3. The umbilical venous partial pressure of oxygen (UV pO_2_) was significantly different between the groups, and it was greater in the oxygen group than the room-air group. The UV HCO_3_ was also significantly different between the groups, again being greater in the oxygen group than the room-air group. In contrast, the UV base excess, umbilical blood gas measurement, and Apgar scores were similar. All scores were ≥7 at one minute after delivery and ≥9 at five minutes after delivery. No maternal and neonatal postoperative complications were reported in the first 24 hours.

## 4. Discussion

This is the first study which focuses on maternal changes in oxygen saturation. Our study demonstrates that the routine administration of supplemental, low-dose oxygen via a nasal cannula during elective cesarean section under spinal anesthesia has potential benefit in terms of a decreased incidence of maternal desaturation. Maternal desaturation definitions have varied from <95% to <90% [11–13], and it has also been defined as a decrease of oxygen saturation of more than 4 percentage points from baseline [14]. We defined maternal desaturation according to the recommendation of O'Driscoll et al. [7] and Van de Louw et al., [9] which was an oxygen saturation of <94% and the event occurring more than 30 seconds after probe checking.

The consequences after arterial desaturation in other groups of patients are severe and life threatening, for example, cardiac arrhythmia, increased risk of wound infection, or memory change. Arterial desaturation may result in attempts to compensate by increasing the respiratory rate and the effort to breath [15]. Respiratory failure may result if these conditions are ignored. Although some previous studies reported that desaturation episodes did not cause decompensation in maternal and fetal status, a continuous pulse oximeter was available for added vigilance in those studies, and treatment was started as soon as desaturation was detected [16, 17].

Supplemental oxygen in obstetrics is widely applied to routine practice because parturients carry a high risk of hypoxemia. Due to the physiological and anatomical changes occurring in pregnancy, maternal oxygen consumption increases, and the functional residual capacity decreases [18]. A significant reduction in respiratory mechanics develops after spinal anesthesia for cesarean delivery [5]. Nevertheless, there are a number of compensation mechanisms. Both maternal and fetal factors enhance the transfer of oxygen across the placenta and thereby prevent fetal hypoxemia. Maternal factors, such as a pregnancy-induced increase in 2,3-diphosphoglycerate, together with the diffusion of acid waste products from the fetus to the mother, induce a reduction in the affinity of the maternal hemoglobin for oxygen, and the fetal hemoglobin has a greater concentration and a higher affinity for oxygen than an adult hemoglobin. Both the maternal and the fetal components promote the release of oxygen from the maternal hemoglobin [19].

The necessity of supplemental oxygen for healthy parturients has been questioned and investigated since a study by Khaw et al. was carried out in 2002. That randomized, controlled trial was conducted on 44 healthy parturients. The authors revealed that hyperoxia after supplemental oxygen induced oxygen free radical activity [20]. In addition, a recent meta-analysis of 10 trials (conducted between 1982 and 2011) of low risk parturients who had undergone elective cesarean section under spinal anesthesia demonstrated that supplemental oxygen was associated with higher maternal and neonatal oxygen levels, and the intervention was neither beneficial nor harmful to the neonatal outcomes [21]. All current knowledge has convinced some anesthetic personnel to change their practice to not giving supplemental oxygen for healthy parturients. The conflicting results of our study compared with the others may arise from methodological differences, that is, the determining of interventions and the main outcomes of interests. Almost all previous studies focused on the effects of oxygen on neonatal outcomes. In our opinion, the measurements of the effects of maternal supplemental oxygen on neonates were far more indirect than the measurements of the effects on maternal outcomes.

Desaturation events seem to be related to maternal hypotension and sedative administration. The overall incidence of maternal hypotension in our study was around 75% (81% in the room-air group and 69.8% in the oxygen group). The incidence of this most common complication after spinal anesthesia ranged from 65% to 80%, depending on the definition of hypotension in each study [22, 23]. Fetal oxygen delivery depends on not only uterine arterial oxygen content but also uterine arterial blood flow. Therefore, maintenance of maternal blood pressure after neuraxial block is also important [12]. Cerebral hypoperfusion following arterial hypotension causes unpleasant signs and symptoms, including nausea, vomiting, and dyspnea, and it may cause desaturation [23]. As for sedated parturient, desaturation after hypoventilation and partial airway obstruction was straightforward. In our study, all desaturated cases were promptly managed. There were no serious complications following desaturation.

One major limitation of our study was that maternal and fetal cord bloods were not investigated to identify reactive oxygen species. These were not routinely available in most institutes. Khaw et al. demonstrated increased reactive oxygen species in those neonates whose mothers received supplementary oxygen during delivery. They also demonstrated a direct relationship between the partial pressure of oxygen in the mother's blood and the amount of ROS in the fetal, umbilical, and venous blood [20]. The clinical effect of the increased reactive oxygen species has not yet been demonstrated. Oxygen given to the mother via a Venturi mask in Khaw's study was higher than the oxygen given via the nasal cannulae used in our study. Hence, the amount of reactive oxygen species, if they increased, should be less. Though UV pO_2_ in the oxygen group was higher than that in the room-air group, the mean difference of 2.8 mm Hg should not be clinically significant. We did not find any clinically significant neonatal outcomes as demonstrated by normal umbilical arterial blood gases and Apgar scores in both groups. However, this cannot confirm long-term safety.

In conclusion, our study had shown the benefit of supplemental oxygen in terms of preventing desaturation in the oxygen group. Nevertheless, every desaturated event could be corrected easily without any significant clinical consequences. If continuous pulse oximeter monitoring is always available, supplemental oxygen in all cases may be optional.

## Figures and Tables

**Table 1:** Perioperative maternal characteristics.

Variables	Room-air group	Oxygen group	*P* value
	(*n* = 163)	(*n* = 162)	
Age (years)	28.7 ± 3.1	28.5 ± 3.3	0.55
Body mass index (kg/m^2^)	27.4 ± 3.0	27.2 ± 3.2	0.74
Indications for surgery			0.44
Previous cesarean section	103 (63.2)	94 (58.0)	
Elective cesarean section	34 (20.9)	45 (27.8)	
Breech presentation	9 (5.5)	9 (5.6)	
Cephalopelvic disproportion	4 (2.5)	1 (0.6)	
Other	13 (7.9)	13 (8.0)	
Baseline hematocrit (%)	35.7 ± 2.8	35.6 ± 3.1	0.79
Amount of preload fluid (mL)	476.7 ± 194.5	467.3 ± 217.0	0.68
Total dose of 0.5% heavy Marcaine and morphine for spinal block	2.2 ± 0.1	2.2 ± 0.1	0.19
Uterine incision to delivery interval (seconds)	143 ± 231	151 ± 75	0.27
Intraoperative hypotension	132 (81.0)	113 (69.8)	0.01
Ephedrine consumption (mg)	20.6 ± 11.0	18.4 ± 11.0	0.10
Norepinephrine consumption (*μ*g)	12.8 ± 9.8	13.1 ± 10.8	0.89
Received sedative drugs	9 (5.4)	15 (9)	0.45
Duration of surgery (minutes)	53.2 ± 13.5	52.8 ± 18.3	0.82
Estimated blood loss (mL)	358.0 ± 125.8	331.0 ± 122.6	0.05
Total fluid administration (mL)	1294.0 ± 341.3	1250.7 ± 342.1	0.26
Intraoperative desaturation	12 (7.4)	0 (0)	0.00

Data presented as mean ± SD or number (%).

**Table 2:** Data of 12 parturients who experienced desaturation.

Number	Age (yr)	BMI	SpO_2_	Baseline Hct (%)	Amount of preload fluid (mL)	Total dose of vasopressor consumption	Estimated blood loss (mL)	During desaturation		Most likely explanation of desaturation
								Concurrent events	Mental status	
1	31	25.7	100	35.6	300	E 30 mg	300	Hypotension after delivery	conscious	Hypotension
2	34	27.6	100	36.5	700	E 30 mg	300	Hypotension after spinal block 2 minutes	conscious	Hypotension
3	35	24.9	96	36	500	E 18 mg NE 36 *μ*g	800	Hypotension before delivery	conscious	Hypotension
4	33	32.7	99	37.7	400	E 18 mg	600	Hypotension after spinal block 7 minutes	conscious	Hypotension
5	32	30.1	100	36	400	E 42 mg NE 16 *μ*g	600	Hypotension before delivery	conscious	Hypotension
6	29	27.6	100	38.4	400	E 6 mg NE 12 *μ*g	600	Hypotension after spinal block 2 minutes	conscious	Hypotension
7	33	29.7	99	31.4	700	E 12 mg NE 8 *μ*g	500	Hypotension after delivery	conscious	Hypotension
8	39	27.0	100	31.2	900	E 30 mg NE 52 *μ*g	500	Hypotension and dyspnea after spinal block 3 minutes	conscious	High block (T2) and hypotension
9	36	26.8	98	33.9	300	E 30 mg NE 12 *μ*g	300	Hypotension and hypoventilation after delivery	Somnolence	High block (T3) and hypotension
10	27	23.9	100	32.6	600	E 6 mg NE 6 *μ*g	700	Received pethidine 25 mg and midazolam 2 mg because of inadequate anesthesia during tubal sterilization	Sedated	Sedation
11	31	27.6	99	34.5	500	E 30 mg NE 8 *μ*g	350	Received ketamine 50 mg after delivery	Sedated	Sedation
12	25	23.8	100	35	650	E 24 mg NE 24 *μ*g	300	Received midazolam 1.5 mg after delivery	Sedated	Sedation

BMI: body mass index; SpO_2_: oxygen saturation before conducting spinal anesthesia; Hct: hematocrit; E: ephedrine, NE: norepinephrine.

**Table 3:** Neonatal data.

Variables	Room-air group	Oxygen group	*P* value
	(*n* = 163)	(*n* = 162)	
Fetal heart sound before spinal block (BPM)	140.3 ± 9.0	140.7 ± 9.4	0.66
Fetal heart sound after spinal block (BPM)	141.6 ± 10.4	140.6 ± 9.4	0.39
Apgar score at 1 minute	9 (7, 10)	9 (7, 10)	0.53
Apgar score at 5 minute	10 (9, 10)	9 (9,10)	0.79
Umbilical cord gas analysis			
UV pH	7.36 ± 0.06	7.37 ± 0.06	0.08
UV pO_2_ (mmHg)	25.57 ± 5.89	28.37 ± 7.91	0.000
UV pCO_2_ (mmHg)	42.91 ± 6.77	43.52 ± 6.14	0.40
UV HCO_3_ (mEq/L)	24.72 ± 3.06	25.39 ± 3.12	0.049
UV base excess	−0.69 ± 3.42	0.07 ± 3.48	0.06
UV O_2_ saturation (%)	45.44 ± 14.92	51.08 ± 17.54	0.002
UA pH	7.30 ± 0.06	7.31 ± 0.06	0.781
UA pO_2_ (mmHg)	21.01 ± 26.61	19.69 ± 6.92	0.542
UA pCO_2_ (mmHg)	52.46 ± 32.86	50.18 ± 9.47	0.397
UA HCO_3_ (mEq/L)	24.92 ± 3.75	25.13 ± 4.29	0.569
UA base excess	−1.42 ± 4.38	−1.16 ± 4.53	0.607
UA O_2_ saturation (%)	24.95 ± 15.90	27.37 ± 15.36	0.168

Data presented as mean ± SD or median (minimum, maximum).

BPM: beat per minute; UV: umbilical vein; UA: umbilical artery.
